# Analysis of Heart Rate Variability in Response to Serious Games in Elderly People

**DOI:** 10.3390/s21196549

**Published:** 2021-09-30

**Authors:** Chun-Ju Hou, Yen-Ting Chen, Mycel Capilayan, Yu-Sian Lin, Min-Wei Huang, Ji-Jer Huang

**Affiliations:** 1 Department of Electrical Engineering, Southern Taiwan University of Science and Technology, Tainan City 710301, Taiwan; cjhou@stust.edu.tw (C.-J.H.); da72b203@stust.edu.tw (M.C.); ma520223@stust.edu.tw (Y.-S.L.); jjhuang@stust.edu.tw (J.-J.H.); 2 Department of Psychiatry, Chiayi Branch, Taichung Veterans General Hospital, Chiayi 000600, Taiwan; hminwei@vghtc.gov.tw

**Keywords:** ECG, HRV, ANS, serious game

## Abstract

As the proportion of elderly people continues to grow, so does the concern about age-related cognitive decline. Serious games have been developed for cognitive training or treatment, but measuring the activity of the autonomic nervous system (ANS) has not been taken to account. However, cognitive functioning has been known to be heavily influenced by the autonomic nervous system (ANS), and ANS activity can be quantified using heart rate variability (HRV). This paper aims to analyze the physiological response in normal elderly people as they play two types of serious games using HRV features from electrocardiography (ECG). A wearable device designed in-house was used to measure ECG, and the data from this device was pre-processed using digital signal processing techniques. Ten HRV features were extracted, including time-domain, nonlinear, and frequency-domain features. The experiment proceeds as follows: rest for three minutes, play a cognitive aptitude game, rest for another three minutes, followed by two reaction time games. Data from thirty older adults (age: 65.9 ± 7.34; male: 15, female: 15) were analyzed. The statistical results show that there was a significant difference in the HRV between the two types of games. From this, it can be concluded that the type of game has a significant effect on the ANS response. This can be further used in designing games for the elderly, either for training or mood management.

## 1. Introduction

The pace of global population aging is continuously increasing. This trend will continue to rise as fertility decreases while the human life expectancy continues to increase, due to better healthcare and advancements in medicine [1]. The World Health Organization (WHO) projected that, by 2050, the world’s population aged 60 years and older is expected to total 2 billion, from 900 million in 2015 [2]. Taiwan is no exception to this trend and is currently experiencing it at an accelerated pace when compared to European countries and the United States [3]. According to the Department of Household Registration of the Ministry of the Interior, the percentage of Taiwan’s population aged 65 years old and above reached 15.28% in 2019 [4]. Taiwan is currently an aged society and may become a super-aged society by 2026 [5]. Thus, Taiwan is facing challenges related to caring for its growing elderly population, especially on their health and well-being.

Mental health is a pressing concern for the elderly population. According to the WHO, over 20% of adults aged 60 and above suffer from a mental or neurological disorder, and 6.6% of all disability among the elderly is attributed to mental and neurological disorders [6]. A study found that there is a significant increase with age in terms of the prevalence of psychiatric disorders in Taiwan [7]. According to the Chief Public Health Officer’s Report in Canada, older adults aged 80 and above tend to be more at risk, with men in this age range presenting the highest rate of suicide [8]. Vulnerable older groups, e.g., people with low income and members of sexual and ethnic minorities, also tend to be overrepresented among those with mental health issues [9]. A major problem in the treatment of mental health issues in the elderly is that they tend to report physical issues compared to psychological issues [10]. One of the major goals in aging research is to investigate methods that can help maintain brain health, cognition, independent living and well-being amongst the elderly.

Aging is heavily associated with changes in the structure and function of the autonomic nervous system (ANS), and these could greatly influence the body’s organ systems. Changes related to aging were found in autonomic nerves and ganglia and ANS-controlled functions [11]. These changes can be associated with age-related cognitive decline. Aging also reduces the efficiency of homeostatic regulation, thus promoting increased tissue damage, rate of morbidity, and mortality [12]. To address some of the effects of aging, especially cognitive decline, some serious games were made for this purpose.

Serious games are often used in studies to improve mood, cognition, and physical and mental well-being for the elderly [13,14,15]. Serious games are games designed for education, simulation, training, or advertising, aside from games’ usual purpose of entertainment. The application of serious games can range from the military, government, education, corporate, and even healthcare [16]. These games have the positive impact towards the well-being of the elderly, such as improving their mood or training their motor and cognitive skills [15].

Playing games requires cognitive abilities. Games can provide stimuli to a person, capable of eliciting cognition and emotion [17]. Since cognition and emotions are closely linked to each other due to being facilitated by the autonomic nervous system (ANS) [18], physiological measures can be measured during gameplay. As aforementioned, aging had been shown to affect the ANS, so there are bound to be changes in how the ANS of an elderly person operates while playing games. Additionally, most studies that use games for training or mood regulation on elderly subjects did not measure physiological measures. Physiological responses also tend to be more reliable, since they can be difficult to control through a person’s awareness [19]. Studying the physiological response while playing, especially in the elderly, may improve game design and biofeedback research designed for this demographic.

One of the commonly used measures of physiological response is heart rate variability (HRV). The heart is connected to the ANS through the vagal and sympathetic nerve fibers. Changes in autonomic nerve activity and the release of hormones influence cardiac activity [20]. Heart rate and HRV have been shown to be a non-invasive and inexpensive method of quantifying the cardiac autonomic function that reflects the regulation of the sympathetic and parasympathetic branches of the ANS [21]. Due to the importance of HRV in health, the standards of measurement, physiological interpretation, and clinical use of heart rate variability was published in 1996 [22].

With this, a system was designed, consisting of serious games designed for elderly subjects, a wearable device designed in-house, and software for recording and a database for storage of the subjects’ data. Analysis of heart rate and HRV features in response to playing different serious games was also performed. The use of physiological measures will provide a more objective outlook on how elderly people with normal cognition (NC) respond to different game types. The subject criteria, experimental design, wearable device, signal processing, feature extraction and statistical analysis are shown in detail in Section 2. The results from the experiment and the discussion and interpretation of these results are in Section 3. Finally, a conclusion is presented in Section 4.

## 2. Materials and Methods

### 2.1. Subjects

To qualify to participate in the experiment, the subject had to be aged between 50 to 85 years. They had to be able to understand the purpose, process, risks, rights, and interests of the research and sign the consent form. Subjects aged below 50 years old or over 85 years old were excluded from the study. People with neuropsychiatric diseases, such as abuse of alcohol or other substances, Parkinson’s disease, epilepsy, etc., were excluded from participating. People with cancer, anemia, uremia, or thyroid dysfunction were also excluded. Severe vision and hearing loss, or any illness or other obstacles that can hinder the person’s ability to cooperate with the researchers was a criterion for subject exclusion.

The subjects were able to withdraw from the trial at any trial stage if they wanted to do so. They could have been withdrawn from the experiment if they were unable to complete a trial, including clinical scale evaluation and board game interactive system. This experiment has been approved by the Ethics Committee Approval (Institutional Review Board (or Ethics Committee) of Taichung Veterans General Hospital Taiwan (protocol code SF18297A-1; 16 January 2019)).

Data from 30 subjects (age: 65.9 ± 7.34; male: 15, female: 15) was used for analysis. Their ages ranged from 51 to 77 years old, with no significant health problems. They were also clinically evaluated for their mental well-being. A mini-mental state examination (MMSE) was used to evaluate the cognitive status among the elderly subjects. The subjects had varying levels of educational attainment, ranging from finishing elementary school to having a master’s degree. Their MMSE scores were carefully evaluated by hospital psychologists that specialized in elderly care. All subjects were characterized as having normal cognition (NC).

### 2.2. Experimental Design

The experiment was conducted in the Chiayi Branch of the Taichung Veterans General Hospital. The experiment room consisted of two testing areas for the subjects to play games. Since all the subjects lived in Taiwan for the majority of their lives, the language used to instruct and play the game was in Traditional Chinese.

**Figure 1:**
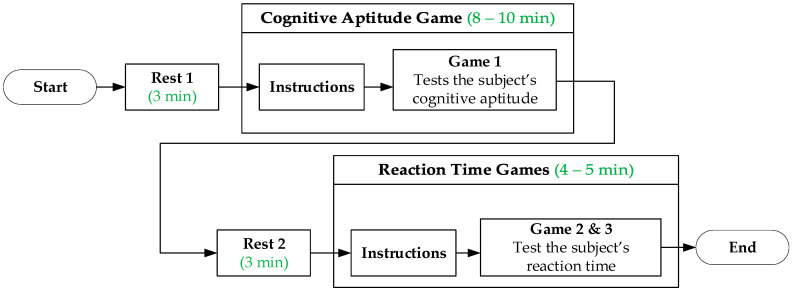
Experiment flow.

The experiment flow is shown in Figure 1. The subject first rests outside the test room. During each rest stage, the subject was asked to sit, relax and close their eyes for 3 min. Although this is shorter than the recommended 5 min, 2 min is the minimum recording time for the HRV features used in this study [23]. Thus, 3 min is more than enough. After resting, the subject was required to walk towards the area where Game 1 was set up and given instructions on how to play the game. The subject was required to rest for 3 min after playing the game, then proceed to another area to play two reaction time games in succession. Game 2 and Game 3 were called Whack-a-Mole and Hit-the-Ball, respectively. After playing, they had to go back to the resting area outside the test room. ECG was recorded during rest and while playing games. The games were played using a personal computer but projected onto a wall for the subjects to have a better view of the games. The cognitive processing that the subject has to do while playing these three games may induce a physiological response that can be measured by heart rate and HRV.

Game 1 is a cognitive aptitude game based on [24], in which the subject needs to answer all questions in each of the five categories: clothing, entertainment, food, housing, and transportation. The gameplay time of the subjects ranged between 8 to 10 min. The game interface was projected to a screen, though it was run using a personal computer. These questions involve memory, attention, executive function, and language.

**Figure 2:**
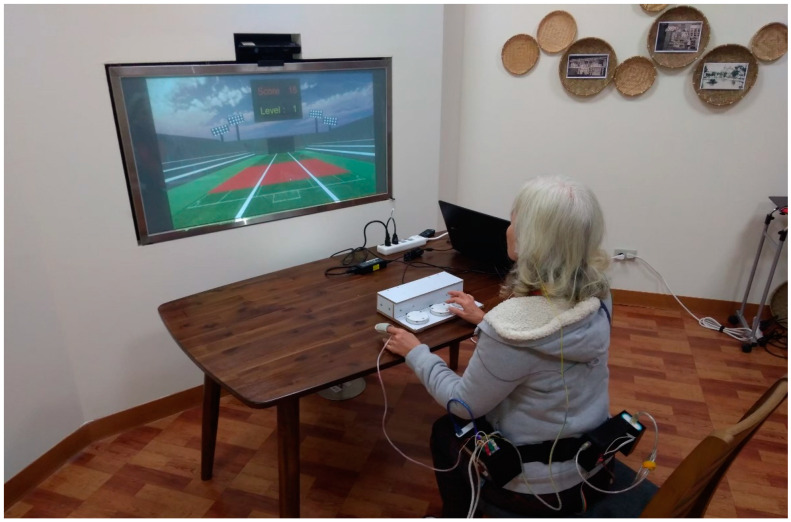
Elderly subject playing Hit-the-Ball.

Whack-a-Mole and Hit-the-Ball are reaction time games, used to test a subject’s accuracy and reaction time [25]. They were designed to gradually get faster and more difficult for the subject to react to the games’ stimuli as time passes. These games require processing speed, which is a person’s cognitive ability defined by how fast they can understand and react to presented information [26]. Gameplay time of the subject ranges between 4 to 5 min. The game was also projected to a larger screen for the subjects to play and run on a personal computer. Figure 2 shows an elderly subject playing one of the games, Hit-the-ball. The ability to recognize the mole or the balls as well as to quickly respond to the stimuli may induce a change in the measured HRV.

### 2.3. Wearable Device

**Figure 3:**
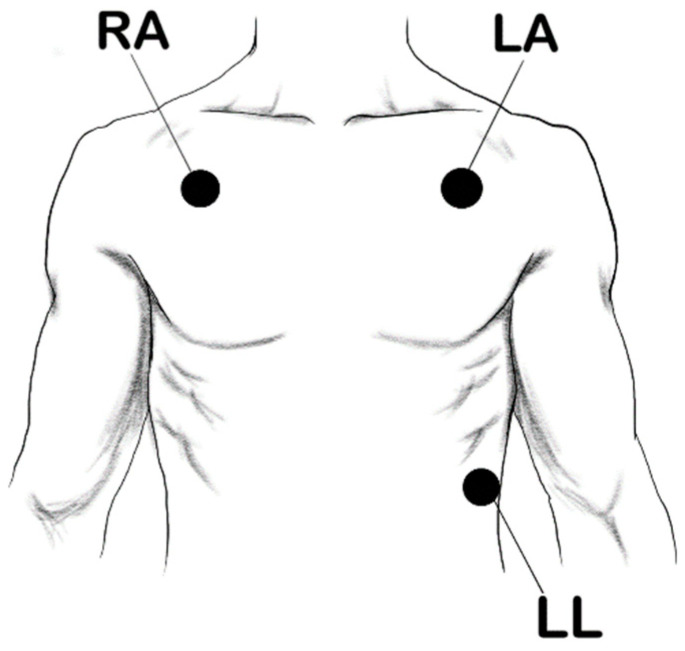
Electrode position using 3 lead ECG.

A wearable device that measures ECG was attached to the subject before the start of the experiment. This device was developed in-house, as it allowed for more flexibility in measuring, storing, and accessing the data. The device was connected to Ag/AgCl patch electrodes placed on the subject, as shown in Figure 3. These electrodes were made of foam and had conductive adhesive hydrogel for better signal conductivity. The ECG signal was obtained through a 3-lead ECG setup, and sampled at 500 Hz. The wearable device could be powered by a power bank. However, since a power bank can only supply +5 V and the device needed −5 and +5 V, a medical-grade DC power converter MOP-05D05A was used for the power management.

Inside the device is the analog circuit module, a digital signal processing (DSP) module, and Bluetooth module. The analog module consisted of a driven right leg circuit (DRL), pre-amplifier, a band-pass filter, a band-stop filter, post-amplifier, and a clamp circuit. The pre-amplifier was implemented with AD620, which is an instrumentation amplifier. The AD620 has a common-mode rejection ratio of 93 dB, which was useful in reducing common-mode noise. It was also used to amplify the signal before any kind of signal processing, since the electrical signal from the ECG sensor is around the millivolt levels. It has a gain of 10, just enough to amplify the signal, while not distorting it. The DRL circuit was added to ensure that the optimum common-mode voltage is applied to cancel out the interference from the subject’s body. To remove unwanted frequencies, the device had two filters, a band-pass filter and a band-stop filter. The band-pass filter was composed of active high- and low-pass filters, each having a filter order of 6, designed with the frequency band associated with ECG signals, which is 0.5 to 150 Hz. The notch filter, with an order of 2, had a reject frequency of 60 Hz and was also designed to remove noise due to the 60 Hz power line in Taiwan. The gains for these filters were 4, 4, and 1, respectively. All filters were Butterworth filters implemented using the Sallen–Key topology. The operational amplifiers used were TL082 and TL084 from Texas Instruments. A post-amplifier with a gain of 2 was used to further amplify the filtered signal, since largely amplifying the signal in the pre-amplifier stage can cause distortion. Finally, the clamp circuit was used to shift the signal level to prevent the signal from being outside the input voltage range of the analog-to-digital converter (ADC) of the microcontroller.

The DSP module used a dsPIC33EP256MU806 microcontroller to convert the analog signals to digital. This microcontroller chip was selected for its low power consumption, multiple sets of ADCs, and UART serial transmission. The sampling rate used for the A/D conversion was 500 Hz, with a resolution of 12 bits. After the conversion, the digital signal was encoded for Bluetooth transmission, with a baud rate of 9600. The HC-05 Bluetooth module was used, since it is designed for transparent wireless serial connection and easy to interface with a personal computer. It has a range of 10 m. The data was then sent to a personal computer for digital signal processing and data analysis.

**Figure 4:**
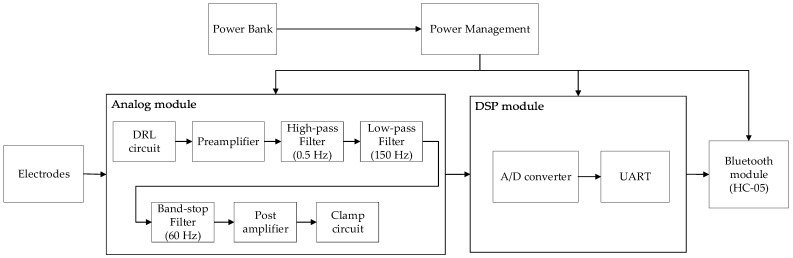
Block diagram of the wearable device.

To receive and record the subject’s data, an application was also developed in-house. MATLAB was used for the development of the application. The platform used in this study was Windows. All the serious games and software used in this study were run using Windows. The block diagram of the device is shown in Figure 4.

The data underwent signal pre-processing, including detrending and filtering, followed by feature extraction. The extracted features were then analyzed using statistical analysis.

### 2.4. Signal Processing and Feature Extraction

The raw ECG signal data undergoes signal preprocessing before feature extraction. The signal was detrended to remove the linear trend or mean value from the dataset. After detrending, the signal is filtered by a Butterworth band-pass filter with a gain of 1 and frequency range from 5 to 40 Hz, from the DSP toolbox in MATLAB to remove baseline drift, white noise, and electromyogram or motion noise. These other types of noise may still be present in the frequency range of the ECG signal and can be introduced during the acquisition of the signal or due to respiration and body movement. The recorded ECG data was then divided into four sections: Rest 1, Game 1, Rest 2, and Games 2 and 3. The time for Game 2 and Game 3 were combined, as they were played in succession, and the subject did not have a resting period in between.

Artifact removal is essential in calculating the interbeat interval (IBI) or RR interval from the ECG data, since HRV features are calculated from this feature. An artifact, even a minor one, can severely skew the calculated HRV [27]. According to the guidelines [22], artifacts such as ectopic beats, arrhythmic events, missing data and noise can affect the calculation of the power spectral density (PSD) of HRV. Artifact removal is especially vital in short-term recordings, as used in the experiment. To remove these artifacts, the tool ARTiiFact [28] was used. It extracts the IBI from ECG, either automated or manual. After extracting the IBI, artifacts were detected using the algorithm proposed by Berntson et al. [29] or by median absolute deviation detection. It also allows for manual artifact detection, in which the user can visually check the signals. To make sure that the calculation of the IBI from ECG was as accurate as possible, we used the automated methods first, then compared and verified their results with manual detection. The detected artifacts were processed through interpolation. Both cubic spline and linear methods were used, as both have their benefits and pitfalls. Linear interpolation is easy to implement and use, but not always suitable for nonlinear data. Cubic spline can provide smooth interpolants but will not work well with sample points that are very close together and have extreme differences in value.

**Figure 5:**
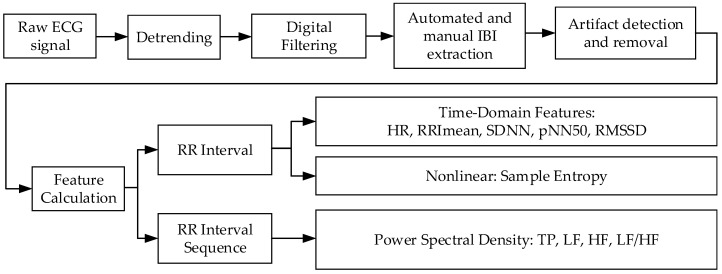
Feature Extraction Process with Data Preprocessing.

After artifact removal, the HRV was calculated using the tool Heart Rate Variability Analysis Software (HRVAS) [30]. It is an open-source tool that calculates HRV features. A total of 16 HRV features were used: 5 time-domain features, 1 nonlinear feature, and 4 frequency-domain features from PSD. The PSD was obtained using autoregressive (AR) power spectral analyses. Figure 5 shows the feature extraction process, including the data preprocessing, and the features extracted to be extracted from the ECG signal.

Table 1 describes the used features in detail. Time-domain features are mainly derived from the detection of the QRS complex from the ECG signal. With each R wave detected, the R–R intervals (the time in-between two successive R waves) are obtained, as well as the instantaneous heart rate. From the calculated R–R intervals, the mean R–R interval (RRI mean), heart rate (HR), standard deviation of NN intervals (SDNN), root mean square of successive differences of NN intervals (RMSSD), and percentage of differences between adjacent RR intervals that are greater than 50 ms (pNN50) can be derived. The calculation of SDNN uses normal beats, which excludes the ectopic beats and other artifacts. It is primarily influenced by parasympatheticaly mediated RSA in short-term recordings. Thus, the high frequency component is more prominent. The pNN50 and RMSSD both strongly correlate to PNS activity, with RMSSD being more accurate for older subjects [23,31].

HRV can be analyzed with nonlinear approaches, and one of these is the sample entropy (SampEn). Sample entropy had been used to measure complexity in a time series. High SampEn values can mean a higher degree of complexity and unpredictability. Lower values, on the other hand, mean higher regularity and predictability [23].

Frequency-domain features are derived from the PSD analysis. The PSD shows power as a function of frequency. There are numerous methods used for calculating the PSD, often classified into parametric and non-parametric methods. Non-parametric methods have high processing speed and have relatively simpler algorithms. On the other hand, parametric methods have smoother spectral components, easier post-processing, and accurate PSD, despite smaller sample sizes. For this study, the autoregressive method, which is a parametric method, was used. The main spectral components of the PSD are VLF (very low frequency), LF (low frequency) and HF (high frequency). The LF band ranges from 0.04 Hz to 0.15 Hz, while the HF band ranges from 0.15Hz to 0.4 Hz. The HF band is associated with the PNS activity, while the LF band is associated with SNS activity [32].

### 2.5. Statistical Analysis

The extracted features were tested for normality using the Shapiro–Wilk test. The Friedman test was used to determine if there were significant differences between the four sessions of the experiment. Then, for the post hoc analysis, Wilcoxon signed-rank test was used after the Friedman test. The calculated significance values were then adjusted using Bonferroni correction. The significance level used for all analyses was 0.05.

## 3. Results and Discussion

### 3.1. Wearable Device

**Figure 6:**
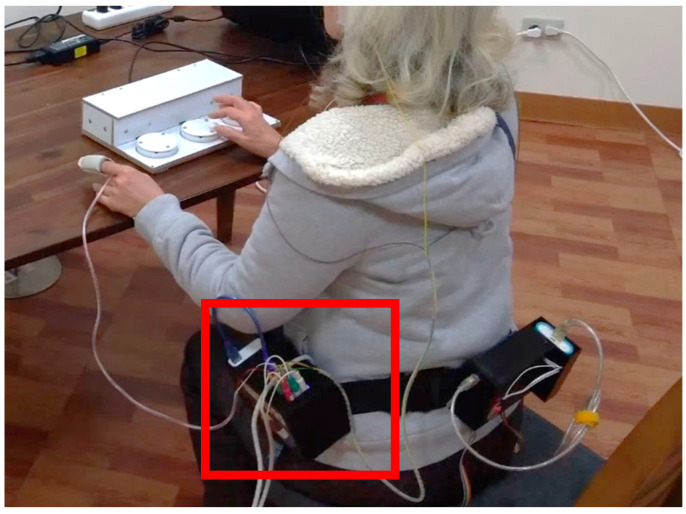
Subject wearing the device. The device discussed in this study is highlighted by the red box.

Figure 6 shows the device being worn by an elderly subject. It measures 15 cm in length, 8 cm in width, and 8 cm in height. It weighs at 193 g, excluding the power bank. It has a larger size compared to other commercial devices, since it was designed to handle different analog modules, such as modules for other biosignals. The power bank used to power the device had a rated capacity of 6400 mAh for and output voltage of 5.1V. With this capacity, the device has a calculated operating time of 18.29 h, as the device has a maximum operating current of 350 mA.

**Figure 7:**
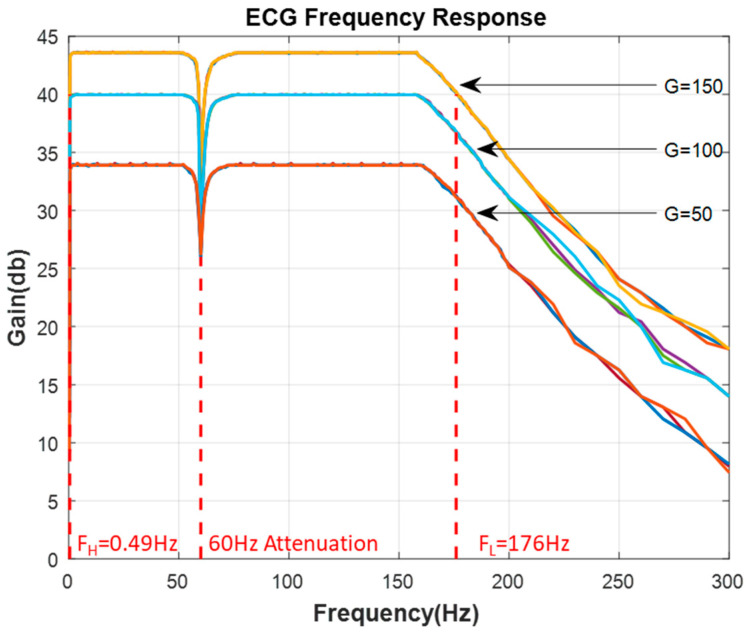
Frequency response of the ECG analog module (Gain (G) = 50, 100, 150).

The ECG analog module was tested for its frequency response with a signal input of 20 mV and input frequencies ranging from 0.3 to 300 Hz. Filter gains of 50, 100, and 150 were used for the frequency response test. These gains were only used for testing, and the actual total gain of the analog module is 320 (10 × 4 × 4 × 1 × 2). The frequency response is shown in Figure 7.

**Figure 8:**
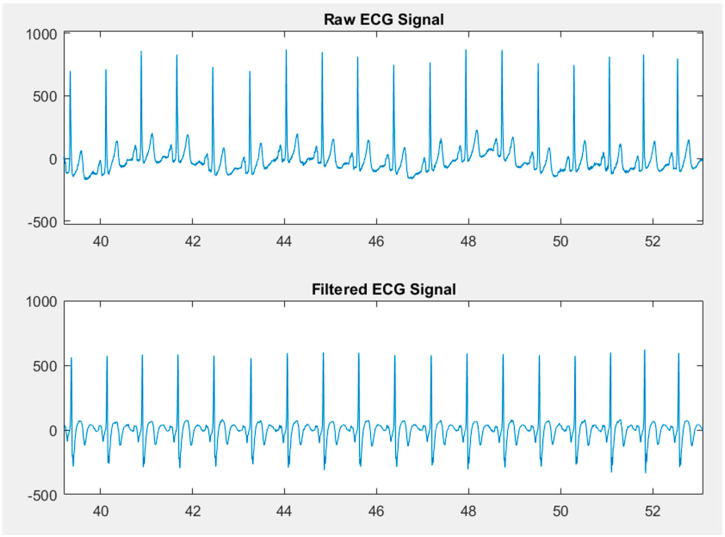
Raw and Filtered ECG signal.

Figure 8 shows the raw ECG signal from the device, and the filtered signal after it was digitally filtered. As seen in the figure, the baseline drift is visibly removed.

### 3.2. Statistical Analysis Results

**Figure 9:**
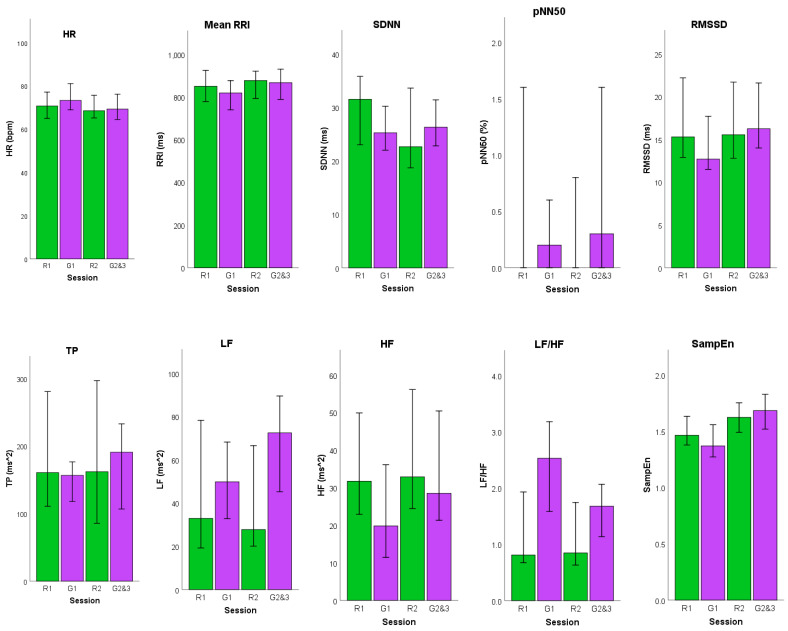
Median values (interquartile range) of the ten features (R1—Rest1, G1—Game1, R2—Rest2, G2&3—Games 2 and 3). The subfigures are bar graph representations of each HRV feature. The green bars represent Rest sessions and the purple bars represent the Game sessions.

Table 2 shows the results from the Friedman test, as well as the median and interquartile ranges of the HRV features. The post hoc comparison for features with significant differences (*p* < 0.05) is shown in Table 3. Figure 9 shows the median and interquartile ranges of the features as a bar graph.

Based on both tables, Rest 1 and Rest 2 did not have any significant difference, with the exception of one feature, SampEn. This is expected, as it shows the recovery to baseline after playing Game 1. Consequently, there was a significant difference between Rest 1, Rest 2, and Game 1 in the majority of the HRV features. This means that there was a significant physiological change in terms of the HRV between the resting sections and playing Game 1. However, the same cannot be said for the second set of games, Games 2 and 3. The majority of HRV features analyzed from playing Games 2 and 3 did not have any significant difference when compared to the HRV from the resting periods, with the exception of SampEn. The reaction time games had a significant difference when compared to Game 1, except for HRV features that show no significant differences between sessions.

Increasing heart rate has been associated with SNS activation, and the significant increase in the heart rate while playing the cognitive aptitude game indicates that the SNS was being activated. This increase in heart rate also correlates with the decrease in the calculated mean RRI. RMSSD, which has been used in numerous studies as an indicator of PNS activity [23,33,34,35], also significantly decreased when the subject was playing Game 1. For Games 2 and 3, there were no significant changes when compared to the two rest periods, but they were significantly different from Game 1. This is further corroborated by the frequency-domain features.

The resting periods have lower LF and higher HF values, indicating that the PNS is activated or dominates over the SNS, which is what usually happens when the body is at rest. The LF values increase while the HF values decrease during gaming sessions. The LF/HF ratio had corroborating results. There was a significant difference between the cognitive aptitude game and the two rest periods. The values increased significantly when the subject was playing the first game and significantly decreased when the subject was at rest. However, the LF/HF ratio did not increase significantly during the second set of games. For SampEn, values were significantly lower during Game 1 compared to Rest 2, as well as with Game 2 and 3. The significant difference between the reaction time games and the resting periods was only observed in SampEn. Additionally, it is only in this feature that Rest 1 had a significant difference with Rest 2. The complexity of the RR interval time series significantly increased from Rest 1 to Rest 2. Additionally, the complexity is significantly higher in Games 2 and 3 than with Rest 1. Decreased values of complexity can be associated with stress [35].

### 3.3. Discussion

In general, the changes in HRV corresponds to other studies using video games. Video games have been shown to induce changes in ANS activity, especially the stress response. HRV features related to PNS activity, such as RMSSD, significantly decreased during periods of gameplay, and returned to baseline levels immediately after [34]. Increased heart rate was observed while playing serious games made in a virtual environment [36]. However, the game genre is a factor to the ANS response. Studies using casual video games have shown that they can improve mood, stress, anxiety, and depression [37]. Studies by Russoniello et al. [38,39] used HRV, and their results show that the measured HRV features coincide with the improved mood of their subjects. However, not all these casual video games are accessible to the elderly. Some serious video games have been used for reducing cognitive decline, but there was low compliance due to the game being less enjoyable and accessible for them [40].

In this study, the changes in the HRV calculated from the four sessions meant that the serious games used were able to elicit different physiological responses. During Game 1, the HRV features that are associated with SNS activity increased, while features associated with PNS activity decreased. The same is true for the resting sections. HRV features associated with PNS activity increased, while the features associated with SNS decreased. However, there were significant differences between the cognitive aptitude game and reaction time games on most of the HRV features. This could mean that the two game types had different effects on the elderly subjects.

SNS activation is usually associated with the stress response. Additionally, PNS activation is associated with relaxation and homeostasis. However, the relationship between the two systems is highly dynamic, and the activation of one system does not always mean that the other is deactivated. However, with the results, the simultaneous increase and decrease in HRV features associated with the SNS and PNS, respectively, most likely indicates that the subjects were experiencing a stress response while playing the cognitive aptitude game. The response was different for the reaction time games. The HRV features did change, but the difference in comparison to rest was not significant. This could be due to several factors. The cognitive aptitude game made the subjects think harder and possibly triggered the SNS activation. On the other hand, the reaction time games might have required lesser mental effort for the subjects, despite the gradually increasing speed. The aesthetics of the user interface and game design could also have an effect. Some stress theories, such as from Lazarus and Folkman [41], state that encountering a potential stressor does not always activate a stress response. The activation is dependent on a cognitive process called stress appraisal. If a stressor is appraised as a threat, it activates a stress response. Otherwise, if it is appraised as a challenge, a stress response is not activated. Thus, the reaction time games could have been appraised by the subjects as a challenge and not a threat. The thinking process required for the cognitive aptitude game could have been considered by the subjects as a threat or a burden, eliciting SNS activation.

The device was able to function was intended. It was able to measure ECG signals and was able to send this data through Bluetooth. Since the device was developed in-house, the recording, storage, and management of data was easier compared to using a commercial device.

## 4. Conclusions and Future Work

In conclusion, we were able to develop a system, comprised of serious games, a wearable device, and software for recording and storage. The developed wearable device was able to measure ECG and send data for HRV analysis. Based on statistical results of the extracted HRV features, the two types of serious games elicited different physiological responses in elderly people with normal cognition. This finding can be used in designing serious games for the elderly, either for training or mood management. A game designer can first make a cognitive aptitude game for training but change the pace of their game at some point, such as adding another game phase that is more action-oriented. Additionally, it can help in making games more accessible, or even encourage their engagement, since physiological signals can be a more objective measure.

Due to the relationship between HRV and ANS activity, significant changes in the calculated HRV coincides with changes in the physiological responses supervised by the ANS. The games used in this study were of two types: cognitive aptitude and reaction time games. Most of the HRV features used had a significant difference compared to baseline while the subject was playing the cognitive aptitude game, and most of the changes imply the activation of the SNS. Recovery was also observed, as the two rest periods have no significant differences for the majority of the features. On the other hand, there were no significant changes in the majority of the HRV features from rest while the subject was playing the reaction time games. Thus, different game types elicited different responses from the subjects. The reasons why could originate in several factors. It could be aspects in the gameplay requiring less effort, or the design of the reaction time games that made the subjects appraise it as a challenge, rather than a threat, which minimized or caused a lack of SNS activation. This could be investigated further.

This study has its limitations. The experiment adopted a sequential order. First, a static cognitive function discrimination test is used. After the test, in Game 2 and Game 3, the test mode is mainly used for the reaction time between both hands and brain. Therefore, it is not suitable to reverse the game sequence. The order of the games was not counterbalanced. Thus, there could be order effects that could affect the results. However, the expected return to baseline in the HRV features was also observed, as presented in the results. In future studies, the order can be randomized to further test and understand the degree of normal people’s recovery from the games.

ECG is one of the mostly used physiological signal due to the high number of features that can be extracted from it. It is also safe to use, non-invasive, and the equipment used to measure ECG is inexpensive to make and produce. Further investigation on the ANS operation and physiological response in elderly people can be done in future studies, especially in biofeedback and serious game design. Biofeedback helps people in making them more aware of their own body by the use physiological signals. It can be through making them aware of their emotions, stress levels or physical condition. Serious games can use the physiological response for designing game mechanics. The difficulty may increase or decrease depending on the calculated HRV features for each gaming session or prompt users to relax for a few minutes before continuing to the next game. The genre or type of games to design can also be taken to consideration, as shown in this study that the game type elicited significantly different response based on their HRV.

For future research, seniors with pre-existing conditions can also be included and may be compared with healthy seniors to see if there are differences in their physiological response. A larger number of subjects could verify or dispute the trends observed in this study. Other physiological signals can also be used in combination with ECG, such as electrodermal activity (EDA), photoplethysmography (PPG) or electroencephalogram (EEG), especially in emotion or stress detection. The wearable device designed and used for this study can be improved further, such as reducing its size and weight. Lower power consumption can also be addressed in the future. Other game designs for the elderly that are focused on training or treatment can be explored, depending on the goal of the game designer. Subjective factors, such as the subject’s opinion on the game can be included to correlate their experience with the measured physiological signals. Aging can affect the function and structure of the ANS, so studies on older subjects may help in providing better healthcare for this age group as well as equip knowledge for the future generation in understanding aging and the elderly.

## Figures and Tables

**Table 1:** List of ECG features used.

Domain	Feature	Description/Significance
Time	HR	Heart rate; SNS activation, HR ↑, PNS activation, HR ↓ [31]
	RRI Mean	Mean R–R interval; Heart rhythm activity [31]
	SDNN	Standard deviation of NN intervals; Overall HRV assessment, PNS activity in short recordings [31]
	pNN50	Percentage of differences between adjacent R–R intervals that are greater than 50 ms; PNS activity index [31]
	RMSSD	Root mean square of successive differences of RR intervals; PNS activity index [31]
Nonlinear	SampEn	Sample entropy; Complexity of R–R interval times series [23]
Frequency	TP	Total power; Variance of R–R interval [32]
	LF	Low frequency (0.04–0.15 Hz) power; SNS activity index [32]
	HF	High frequency (0.15–0.4 Hz) power; PNS activity index [32]
	LF/HF	LF to HF ratio; SNS and PNS balance [32]

**Table 2:** Friedman Test Results.

Features	Rest 1	Game 1	Rest 2	Games 2 and 3	*p*-Value
HR (bpm)	70.75 (17.25)	73.35 (15.70)	68.55 (15.20)	69.30 (13.93)	0.000 *
RRI (ms)	850.05 (214.65)	818.50 (168.68)	877.10 (190.08)	866.85 (169.65)	0.000 *
SDNN (ms)	31.50 (17.08)	25.25 (10.93)	22.65 (25.90)	26.30 (12.80)	0.066
pNN50 (%)	0.00 (2.73)	0.20 (1.83)	0.00 (1.80)	0.30 (2.15)	0.214
RMSSD (ms)	15.30 (13.30)	12.70 (8.35)	15.55 (12.88)	16.25 (11.13)	0.001 *
TP (ms^2^)	160.76 (250.29)	156.69 (114.62)	162.09 (301.72)	190.89 (174.28)	0.632
LF (ms^2^)	32.93 (92.83)	49.83 (73.60)	27.79 (74.87)	72.47 (79.15)	0.106
HF (ms^2^)	31.72 (48.50)	19.83 (33.07)	32.89 (52.42)	28.50 (40.32)	0.000 *
LF/HF	0.81 (2.12)	2.53 (2.22)	0.85 (1.91)	1.68 (2.16)	0.000 *
SampEn	1.46 (0.39)	1.37 (0.42)	1.62 (0.38)	1.68 (0.39)	0.003 *

* *p* < 0.05, Data are expressed as median (interquartile range).

**Table 3:** Post hoc comparison for features with significant differences.

Features	Post Hoc Analysis
HR (bpm)	(2) > (4); (2) > (1); (2) > (3)
RRI (ms)	(2) < (4); (2) < (1); (2) < (3)
RMSSD (ms)	(2) < (4); (2) < (1); (2) < (3)
HF (ms^2^)	(2) < (4); (2) < (1); (2) < (3)
LF/HF	(2) > (4); (2) > (1); (2) > (3)
Samp En	(2) < (4); (2) < (3); (1) < (3); (1) < (4)

(1)—Rest 1; (2)—Game 1; (3)—Rest 2; (4)—Games 2 and 3.

## Data Availability

Data are available upon request due to ethical and privacy reasons. The data are not publicly available due to privacy concerns of the subjects.
